# Magnetoencephalography: Fundamentals and Established and Emerging Clinical Applications in Radiology

**DOI:** 10.5402/2013/529463

**Published:** 2013-08-12

**Authors:** Sven Braeutigam

**Affiliations:** Oxford Centre for Human Brain Activity, Department of Psychiatry, University of Oxford, Warneford Hospital, Oxford OX3 7JX, UK

## Abstract

Magnetoencephalography is a noninvasive, fast, and patient friendly technique for recording brain activity. It is increasingly available and is regarded as one of the most modern imaging tools available to radiologists. The dominant clinical use of this technology currently centers on two, partly overlapping areas, namely, localizing the regions from which epileptic seizures originate, and identifying regions of normal brain function in patients preparing to undergo brain surgery. As a consequence, many radiologists may not yet be familiar with this technique. This review provides an introduction to magnetoencephalography, discusses relevant analytical techniques, and presents recent developments in established and emerging clinical applications such as pervasive developmental disorders. Although the role of magnetoencephalography in diagnosis, prognosis, and patient treatment is still limited, it is argued that this technology is exquisitely capable of contributing indispensable information about brain dynamics not easily obtained with other modalities. This, it is believed, will make this technology an important clinical tool for a wide range of disorders in the future.

## 1. Introduction

Magnetoencephalography (MEG) is a noninvasive technique for recording brain activity. MEG was first introduced to the scientific community in 1972 [1], and it has undergone substantial technological advances ever since. Modern multichannel, whole-head systems provide reliable, fast, and patient friendly scanning that is increasingly being used for clinically oriented research into a wealth of mental disorders and abnormal conditions, such as adult and pediatric epilepsy [2–6], autism [7, 8], schizophrenia [9], Williams syndrome [10], Landau-Kleffner syndrome [11], Alzheimer's disease [12, 13], depression [14], attention deficit hyperactivity disorder [15, 16], and dyslexia [17]. Moreover, MEG has been used to study neuronal change and reorganization following stroke [18], head trauma [19], and drug administration [20]. MEG research centers now exist in many countries, with perhaps Japan, USA, Germany, UK, and Finland leading in terms of total installations. MEG has been approved for clinical evaluation by FDA (Food and Drug Administration) and Medicare in the USA, where many insurance companies presently are covering MEG scans in patients with epilepsy, intracranial neoplasia, and vascular malformations [6]. Typically, MEG scans have to be coordinated on a case-by-case basis requiring efforts on the part of the MEG center, the patient's doctors, and the patient. Centers elsewhere are beginning to accept MEG scanning request from doctors and other healthcare professionals, although practice and support may vary considerably between countries.

Despite increasing availability of high-end scanners, however, the dominant clinical use of MEG currently centers on two partly overlapping areas, namely, localizing the regions from which epileptic seizures originate, and identifying regions of normal brain function in patients preparing to undergo brain surgery. As a consequence, many radiologists may not yet be familiar with this powerful technique. The purpose of this review is to provide the reader with an introduction to MEG fundamentals and relevant analytical techniques including an exposition of modern approaches to artifact correction, an issue of particular importance to clinical studies. Although extensive, this paper does not attempt to discuss every possible clinical application of MEG. Instead, emphasis is given to recent developments in two broad areas, namely, epilepsy and autism. The former area is rather well established implying a reasonably defined role of MEG in diagnosis, treatment, and patient management, whereas the latter is an emerging field for MEG, reflecting a growing trend in using neuroimaging in the study of developmental disorders.

## 2. MEG Technology

**Figure 1:**
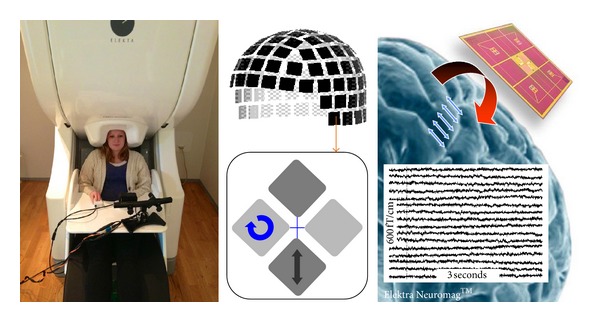
The MEG scanner at the Oxford Centre for Human Brain Activity. Left: Shown is a typical set up, where the subject is seated upright under the scanner. A button box is used to record behavioral responses and an eye-tracker supplies additional psychometrical data. Measurements in supine position are possible, and a doctor or nurse can stay within the magnetically shielded room in order to monitor the patient if needed. Middle: The brain magnetic fields are recorded with a helmet-shaped array of 102 SQUID devices featuring 3 pick-up coils each (306 channels in total). Two first-order gradiometers measure two orthogonal spatial gradients of the magnetic field in longitudinal and latitudinal directions, respectively. These channels are most sensitive to the tangential neuronal currents in the region below the device. A magnetometer coil (black rectangle) measures the magnetic field and is sensitive to deeper sources. Right: Typically, the cortex-to-detector distance is 4-5 cm. The primary output of the scanner is a trace for each channel representing the magnetic field or gradient as a function of time (for presentation, only 16 channels are shown; brain image courtesy of Elekta Neuromag OY, Helsinki).

MEG is based on the detection of the magnetic fields that are generated by the currents flowing in neurons. Unlike positron emission tomography (PET) or functional magnetic resonance imaging (fMRI), MEG does not rely on secondary effects induced by brain activity but directly measures the magnetic fields primarily generated by postsynaptic neuronal ionic currents. In contrast to its closest cousin, the electroencephalogram (EEG), MEG signals are reference-free and essentially unaffected by conductivity differences on the magnetic flux, providing an almost undistorted view of brain activity, and simplifying data analysis and interpretation of patterns. MEG is mainly sensitive to magnetic fields generated in the cerebral cortex, but modern, whole-head systems with multiple sensor configurations can detect activity in subcortical regions. Typically, neural magnetic field changes are extremely small, ranging from 10^−14^ T, or even less, for evoked fields to approximately 10^−12^ T during interictal epileptic spikes [21, 22]. By comparison, a typical fMRI scanner operates at 1.5 T, and the Earth's magnetic field is approximately 10^−4^ T. Currently, the only practical and competitive detectors capable of recording such small field changes are superconducting loops that are coupled into superconducting quantum interference devices (SQUID) that respond to the changes in magnetic flux through the loops [23, 24]. An MEG scanner is illustrated in Figure 1.

The loop configuration determines what the detector measures; for example, a simple planar coil measures the change in the magnetic field perpendicular to the loop plane, and a figure-of-eight-shaped coil measures the change in the magnetic field gradient. An analogy is in order. The loop (coil) can be compared to a lens forming an image dependent on the focal length, where the SQUID transforms the magnetic fields into electrical voltages much like a camera sensor transforms an optical image. A typical MEG imaging system has several hundred sensors surrounding a head shaped recess in a liquid helium-filled Dewar (cryogenic temperatures are required for SQUID operation). The whole instrument is housed in a magnetically screened room that is required to reduce the ambient magnetic field noise. The best instruments currently available achieve noise levels approaching 10^−16^ T per square root of the bandwidth over which the signals are recorded. The temporal resolution of MEG is less than a millisecond, which is sufficient to trace even the fastest neuronal processes. The spatial resolution of MEG depends on signal quality and can be as low as a few millimeters for appropriate source models, which is in the range of subdural electrode strips and grids [6, 25].

There are subtleties associated with the sources that are detected. Even with modern scanners, the fields emanating from single nerve cells are too weak to be measured outside the head. Similar to EEG, this implies that detectable field changes require large numbers (few thousand to a few ten thousands) of neighbouring cells to carry aligned currents of suitable strength and duration to allow for spatial and temporal and spatial summations. It is commonly assumed that these conditions are met through postsynaptic currents rather than currents associated with action potentials or other electrobiochemical processes [26–28] and [29, Chapter 1]. MEG is technically challenging; however, this technology has some significant advantages. It is a passive and contactless measurement that is completely non-invasive, easy to prepare, and minimally demanding on the subject. The instrument is silent in its operation, where the MEG detector records the brain activity continuously as the subject performs tasks required by the investigator. Longitudinal studies are ethically possible. MEG is capable of providing meaningful results on its own, although information extracted from other brain imaging techniques and coregistered with the MEG can be used to guide analysis and interpretation.

## 3. Analytical Approaches

The primary outputs provided by any magnetoencephalographic scanner are readings of the variation in time of the magnetic fields produced by electrical activity in the brain and detected over the head. These signals are analyzed in various ways, correlated with experimental conditions and interpreted within the framework of a given clinical investigation, where often findings obtained from other imaging modalities are considered or even integrated into the analysis. A plethora of analytical techniques to extract information from the MEG signals have been developed, and new methods continue to emerge rapidly. Currently, the abundance of approaches implies an absence of standard protocols for MEG analysis, but there is a clear drive towards standardization, and guideline papers are beginning to emerge [30]. Despite the variety and partial incomparability of methodologies, however, certain approaches have crystallized that are used fairly consistently across clinical studies.

### 3.1. Event-Related Fields

Event-related fields (ERF) are direct analogues of the perhaps more familiar event-related potentials (ERP). Event-related approaches rely on repeatedly presented auditory, visual, tactile, electrical, or other stimuli. The appearance of each stimulus marks a point in time, usually denoted as stimulus onset, with respect to which an epoch of data is defined. The signal observed in the post-stimulus interval is assumed to be independent of stimulus repetition but to be contaminated by random brain signals considered to be noise. This assumption is often justified but may have limited validity in studies of higher order function. Within the assumption of independence against repetition, the stimulus-specific neural response can be recovered from the measured signal by averaging the data with respect to stimulus onset. The resultant waveform is usually called an evoked response. The process of averaging may be extended over several subjects to yield grand mean waveforms. Typically, the stimulus and subject dependence of peak amplitudes and latencies of the evoked responses allow some insight into their functional significance [31].

### 3.2. Time-Frequency Analysis

Viewing brain activity as an oscillatory phenomena, there is a long history of studying fundamental brain rhythms such as theta (4–7 Hz) or alpha (8–13 Hz) waves that, under certain conditions, may indicate general states of the brain; for example, increased alpha is often seen when subjects close their eyes [32]. Robust algorithms are now available to analyze neurophysiological signals in terms of oscillations that vary in frequency and time. Typically, transient oscillatory brain activity is considered in the context of well-defined stimuli and is identified as either an evoked oscillatory response appearing at the same latency and phase in each trial or an induced oscillatory response appearing with a jitter in phase from one trial to another and therefore not detectable in the averaged data. Both types of response are assumed to provide information about brain dynamics beyond what can be extracted from the conventional evoked response, which includes all frequencies [33, 34].

Oscillations at higher frequencies in the beta (15–30 Hz) and gamma (>30 Hz) bands have received much attention in recent years. Experimental data reveal that synchronization of activity in these bands is involved in processes such as working memory, semantic processing, and sensory-motor integration and that synchronization may be altered under pathological conditions [35, 36]. Theoretical considerations suggest that synchrony of oscillations can facilitate the coordinated interactions of large neuronal population distributed within and across distinct regions of the brain, where interneuron networks are believed to play an important role [37, 38].

Of particular relevance to clinical application is event-related desynchronization (ERD). ERD refers to the well-known phenomenon that certain events or stimuli can desynchronize or even block parts of the ongoing brain activity. These induced changes can be detected using a technique employing band-pass filtering, squaring, and averaging in order to obtain a measure of the oscillatory power in a given frequency band of interest. ERD is then quantified relative to the oscillatory power obtained from a reference interval. The ERD measure may become positive dependent on the nature of task and stimuli, in which case one uses the term event-related synchronization (ERS). ERD (ERS) may be considered to be due to a decrease (increase) in synchrony of the underlying neuronal populations and is recognized as a robust marker of normal and abnormal neuronal processes in clinical applications of EEG and MEG [39].

### 3.3. Source Estimation

Most relevant to clinical MEG is source estimation, that is, the identification of the brain areas generating a given signal recorded by the detectors (also known as magnetic source imaging, MSI). Source estimation is ultimately based on the theory of electromagnetism which is governed by 4 equations, known as Maxwell's equations, which relate the spatial and time derivatives of the electric and magnetic fields to the charges and currents present within a given volume defined by its geometry, electric permittivity, and magnet permeability [40]. In the case of electrophysiological signals, the useful frequency spectrum is typically below 2 kHz, and most studies deal with frequency between 0.05 Hz and a few hundred hertz. As a consequence, one can disregard the time derivatives yielding the quasi-static approximation

(1) $$\begin{array}{c} B\left(r\right)\sim \int L\left(r,r^{\prime }\right)\times J\left(r^{\prime }\right)dr^{\prime }, \end{array}$$

also known as Biot-Savart's law. The rule relates the current flow *J* at location *r*′ within a given source volume to the magnetic field *B* observed at position *r*. The integral (continuous sum) runs over the volume containing the currents, where the lead field *L* establishes a link between currents and magnetic field taking into account volume geometry and properties. Typically, one associates the magnetic field with the actual measurement, in which case the lead field is also dependent on the type, position, and orientation of the detector used. Then, the physical interpretation of the lead field is that it represents the sensitivity pattern of a given detector and a given source volume [21, 41].

Conceptually, the law constitutes a forward problem; that is, knowing the currents and the properties of the embedding medium, one can calculate the magnetic field in an analytical or numerical fashion. In contrast, every source estimation procedure has to face the so-called inverse problem, that is, the mathematical transformation that uses the observed magnetic field as input and determines the sources of the signals. Here, there is a problem that is shared with many algorithmic procedures—the inverse problem in MEG is deeply mathematically ambiguous [41, 42]. No matter how high quality the data is and how many measurement channels one uses, there can always be silent albeit neurophysiologically relevant currents in the brain that are not recorded by the detectors. The MEG inverse problem is approached pragmatically by simplifying the description of the sources or by adding additional physiologically reasonable assumptions. The forward model is always needed for source estimation, and it is commonly assumed that the current flow in the head volume can be expressed as the sum of a primary or impressed current due to the electromotive forces associated with biological, mainly synaptic, activity in neural tissue and a passive current flow that results from motion of extracellular charge carriers [43]. Consider

(2) $$\begin{array}{c} J\left(r\right)=J_{\text{impressed}}\left(r\right)+J_{\text{passive}}\left(r\right). \end{array}$$

#### 3.3.1. Equivalent Current Dipoles

The arguably simplest and, until recently, most commonly used source estimation technique is the use of equivalent current dipoles (ECD). An ECD can be viewed as the spatial average of all impressed currents within an area of the brain assuming that the area's spatial extent is small compared with the distances to the detectors. Each equivalent dipole is characterized by 6 parameters describing the dipole's position, orientation and strength within the head. In general, a nonlinear iterative algorithm is applied in order to find the parameters best describing the data measured. In each iteration step, the magnetic fields generated by the dipoles are compared with the fields measured by the detectors, and the parameters are adjusted to minimize the field difference quantified by a cost function. Typically, a single homogenous sphere is used to approximate the head volume, although other conductor models have been used such as ellipsoids, several spheres each fitted to the local curvature of the head, or realistically shaped geometries based on individual structural scans.

In case of the homogenous sphere, the magnetic forward problem becomes independent of tissue conductivities and the spherical symmetries imply that radially oriented current dipoles become hidden. Thus, this source model can only account for tangential dipoles assumed to be primarily located in the walls of cortical fissures and sulci but not in the convexial cortex [21, 41, 44]. ECD approaches vary according to the number of dipoles employed (one dipole being the most common choice), the number and type of parameters allowed to change during fit (e.g., fixed position with rotating orientation and time-varying strength), and the nature of searching or scanning algorithm used to fit parameters to data.

#### 3.3.2. Imaging Methods

Imaging approaches to the inverse problem explore quasi-continuous estimates of activation by dividing the brain volume into distinct voxels and allocating 3 orthogonal equivalent current dipoles to each voxel. Several thousand voxels are usually employed approximating any arbitrary spatial distribution of post-synaptic currents in the brain. The inverse problem in this case is linear, since the source locations are known leaving only source amplitudes to be estimated. The problem, however, is severely underdetermined given that the number of detectors is small compared to the number of voxels, and regularization is required in order to restrict the range of allowable source currents while simultaneously minimizing the difference between measured and predicted magnetic fields.

Symbolically,

(3) $$\begin{array}{c} {\text{minimum}}_{\left\{J\right\}}\text{:}\;{||B-L\times J||}^{p}+\lambda {||W_{\text{model}}\times J||}^{p}, \end{array}$$

where ||⋯||^*p*^ denotes a norm (roughly, strength) of the quantity enclosed. In other words, one estimates for all voxels those current amplitudes that best explain the data and keep a chosen model term, also known as a penalty term, small. Commonly, the voxels are not uniform throughout the cranium but restricted to the grey matter or more specifically to the cerebral cortex. In line with the assumption that currents in the dendritic trunks of pyramidal cells predominantly contribute to external fields, the dipole orientation may further be constrained to be perpendicular to the local cortical surface. The parameter *λ* controls the amount of regularization, and its optimal value is usually determined in a data-driven fashion using an appropriate heuristic approach. The weight matrix *W*_model_ is used to assign additional properties of the inverse solution such as focal sources. In essence, the weight and the norm parameter *p* together define the type of imaging solution, each of which has its merits and disadvantages [44–50]. The most common choices of norms and weights are summarized in Table 1.

#### 3.3.3. Beamforming

Beamforming approaches to the inverse problem aim at discriminating between signals originating from a location of interest and those generated elsewhere in the brain. Similar to imaging techniques, one assumes that the MEG measurement can be modeled by a set of fixed current dipoles distributed throughout the brain. In contrast to imaging techniques, however, one does not attempt to solve a set of underdetermined equations using norm constraints. Instead, a beamformer estimates the neural activation at each voxel independent of all other locations by means of a spatial filter. Ideally, such filter *F* has complete spatial specificity zeroing the lead field at all locations except the chosen one

(4) $$\begin{array}{c} F\left(r_{0}\right)\times L\left(r,r^{\prime }\right)=\{\begin{array}{cc} 1, & r^{\prime }=r_{0},\\ 0, & r^{\prime }\neq r_{0}, \end{array} \end{array}$$

where *r*_0_ and *r*′ are locations with the source volume and *r* refers to the location of measurement. Once the filter has been estimated, the neuronal current at position *r*_0_ can be recovered from the magnetic fields detected by the sensor array

(5) $$\begin{array}{c} J\left(r_{0}\right)=F\left(r_{0}\right)\times B. \end{array}$$

This expression is commonly interpreted as a *virtual electrode*; that is, one gains knowledge about a current as if an electrode was attached to the location of interest. In practice, it is generally impossible to have complete attenuation in the stop-band, that is, locations other than the one of interest, and one aims for designing a filter that is optimal in some sense [44, 51].

Arguably the most influential guiding philosophy for designing an optimal filter is the linear constrained minimum variance approach, or LCMV for short [51]. The idea is to determine a filter that minimizes the variance of the source at a given location while satisfying the linear response constraint *F* × *L* = 1 ensuring that the signals of interest pass by the filter. Minimization of source variance, which is a measure of current strength, optimally allocates the stop-band response of the filter to minimize the contribution to the output due to signals generated by strong sources in the stop-band. The mathematical apparatus of beamforming relies on the assumption that the dipole sources generate mutually uncorrelated brain activity. Correlated activation cannot be reliably disambiguated from correlated signals at the sensor level due to volume conduction and field propagation. In other words, the sensor readings might be correlated because underlying activations are correlated or the field generated by a source is detected by more than one sensor at the same time. The assumption that brain activity is uncorrelated across regions might not be physiologically justifiable; however, it has been shown that beamformer source estimates are, to some extent, robust against correlated sources [44, 51, 52].

A related approach is known as hardware beamforming exploiting the physical properties of certain detectors types. Sensors like planar gradiometers employ two coils measuring orthogonal magnetic field gradients (spatial variation) at each sensor location. The root-mean-square of the two readings can be interpreted as the strength of a current dipole located directly below each gradiometer, yielding a sensor-level map of brain activation that can be projected onto the cortical surface. The method is easy to implement and might be applicable even if the scanner does not have sensors directly suitable for hardware beamforming [53].

#### 3.3.4. Summary: Source Estimation

The researchers and clinicians can now draw on a wide range of source reconstruction methods, often available in form of commercial and open software packages. The source images obtained from different algorithms are often broadly consistent and may, in favorable circumstances, offer meaningful insights into functional anatomy at the centimeter level. However, no general source imaging method is currently available, where the results may depend on the data and method of analysis. In particular, the spatial extent of source images may reflect the properties of the estimate rather than the true extent of the neural current. Statistical inferences based on source estimates need to be judged with care, as significance does not necessarily imply significantly nonzero real primary current in a voxel [54]. In any case, the interpretation of source estimates should include consideration of the nature of the underlying algorithm.

## 4. Artifacts and Artifact Removal

**Figure 2:**
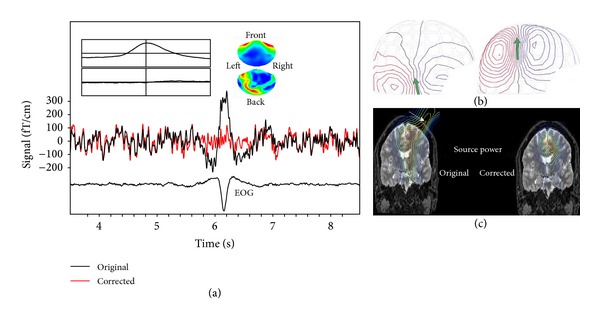
Artifact removal. (a) Shown are data from one channel before (black) and after (red) ICA-based correction for eye-blink artifacts identified by EOG activity (bottom trace). The inset illustrates the 2-dimensional artifact space used for projection (time-courses and spatial topographies). The first dimension is dominant. Note the artifact has similar features as epileptic discharges in that it involves several MEG channels, has sharp peaks, and stands out from ongoing background activity (see also [55]). (b) Equivalent current dipole modeling of an evoked somatosensory response in a child before (left) and after (right) SSS-based movement correction. After correction, the ECD assumes a physiologically plausible location (courtesy of Elekta Neuromag OY, Helsinki). (c) Brain activity can be localized accurately despite strong artifacts caused by DBS electrodes (right). Before beamforming-based correction, strong, nonphysiological interferences outside the brain are observed (left, adapted from [56]).

The high sensitivity of MEG implies that the recordings are sensitive to artifacts originating from a variety of physiological and environmental sources. This issue is not trivial and can become a serious challenge in certain patient populations due to, amongst others, heightened physiological responses, lack of control over movements, and magnetic materials necessary for diagnosis and treatment [4]. In general, the impact of artifacts may be reduced or controlled to some extent through careful preparation and experimental design, and exclusion of the most affected data may facilitate analysis and interpretation. However, it is impossible to exploit clinical data fully without advanced approaches to signal processing that can reliably separate brain signals and artifacts (Figure 2 illustrates the concepts discussed in this section).

### 4.1. Physiological Artifacts

Arguably the two most dominant artifact sources are the heartbeat and eye blink. Although these physiological artifacts can have rather large amplitudes, signal separation is challenging, as the artificial signals may resemble activity originating from neuronal sources. Regarding the eye, normal, automatic blinking occurs between about 5 and 20 times per minute and causes magnetic fields over predominately frontotemporal regions that can be much larger than the magnetic fields of spontaneous and evoked brain activity. The primary artifact is generated by current flow between the eye and scalp and by excitation of ocular muscles controlling eye and eyelid movements. The artifact time course recorded by MEG or EEG during a spontaneous blink corresponds to the eyelid motion reaching a closed position on average 120 ms after the blink is initiated and before returning to a fully open position approximately 240 ms after closure. In addition to the direct artifact fields, blink related neuronal activity lasting up to several tenths of a second is also often observed over posterior parietal and frontal cortices as well as other areas of the brain associated with higher-order processing [57, 58]. For artifact correction, one generally obtains an independent record of the artifact by measuring the electrooculogram (EOG) with electrodes placed close to the eyes.

Regarding the heart, electrical currents moving through the organ during a heartbeat give rise to magnetic fields that are, over the chest, between two and three orders of magnitude larger than the brain magnetic fields. The time course of the cardiac artifact closely corresponds to the propagation of electrical activity through the heart muscle. Each heartbeat begins with an impulse from the sinoatrial node, the heart's main pacemaker, activating the upper chambers (atria). This activation is seen as the P wave (duration about 80 ms) in the electrocardiogram (ECG). Next, the electrical current flows down the heart and activates the lower chambers (ventricles) giving rise to a characteristic wave known as the QRS complex (80–120 ms). Subsequently, the electrical current spreads back over the ventricles in the opposite direction generating a recovery wave commonly denoted by the letter T (160 ms). Typically, these currents flowing in the heart are observable to varying degrees in MEG and EEG recordings causing artifacts that may resemble epileptic spikes and slow waves. It is generally assumed that secondary artifacts due to heart beat related blood pulsation, body movements (ballistocardio-effects), and susceptibility changes are negligible but may exist in MEG and EEG recordings if the lungs, skin, or blood vessels contain magnetic particles [59, page 162] and [60]. For artifact correction, one generally obtains an independent record of the artifact by measuring the ECG with electrodes placed on both arms close to the wrists.

Various approaches have been suggested in the past to correct for these artifacts, often but not exclusively utilizing equivalent current dipoles to model nonneuronal aspects of the artifacts, for example, the retina's electrical charge [61]. Despite merits, however, those methods are increasingly being replaced by signal projection-based approaches. Projection methods are broadly analogous to obtaining a 2-dimensional shadow from a 3-dimensional object where the direction of illumination determines which features are visible in the projection. Mathematically, the data are represented as points (also called vectors) in a space of dimension *n* equal to the number of measurement channels. Each *n*-dimensional vector corresponds to one sampled time point. Then, the set of new vectors, *V*, is calculated to form a basis for the unwanted signal features, that is, the artifact to be removed. In other words, the set *V* spans a subspace that is, as far as possible, occupied by the artifact but not the neurophysiological signals of interest. An artifact-free (clean) measurement is readily obtained through projection

(6) $$\begin{array}{c} M_{c}=\left(\underline{1}-V\times V^{+}\right)\times M, \end{array}$$

where *M* denotes the measurement matrix of dimension *n*  × (number of time-points). $\underline{1}$ denotes the *n*-dimensional identity matrix and *V* ^+^ is the pseudoinverse of *V*, which has dimension *n* × *k*, where *k* is less than *N* [62]. Dependent on the artifact to be removed, *k* typically assumes a value between 1 and 3.

Perhaps the most powerful method to determine the artifact subspace *V* is a type of blind source separation (BSS) known as independent component analysis (ICA). ICA is entirely data driven and assumes that the observations *M* constitute a linear superposition (mixing) of statistically independent source signals. The number of independent sources, *s*, is assumed to be the most equal to the number of measurement channels. A frequently quoted analogy is the “cocktail party problem.” In this scenario, party guests hold multiple, simultaneous conversations recorded using microphones located at fixed positions around the room. Knowing the number of speakers (*s*  ≤ number of microphones), ICA allows recovering of the individual conversations by unmixing the recordings

(7) $$\begin{array}{c} S=U\times M, \end{array}$$

where the number of rows of *S* is equal to the number of speakers. Each row of *S* contains the sound from one speaker only. The number of columns of the un-mixing matrix *U* is equal to the number of microphones, and each element of *U* can be interpreted as a weight determining how much an individual speaker's voice contributed to a given microphone [63, 64]. Using this analogy, the task is then to identify the “speakers” who deliver the artifact and guide them out the room.

Over the last 7 years or so, several algorithms have been published in order to address this issue in an automated fashion. The main feature that distinguishes the different approaches is whether or not an independent EOG and/or ECG recording is needed for artifact identification.  Table 2 shows a brief overview of some of the relevant studies.

A recent study in 26 healthy volunteers compared various artifact detection metrics as well as different flavors of ICA and other blind source separation techniques [70]. In general, the authors found automated artifact removal methods practicable, where satisfactory results can be obtained in most cases irrespective of the detailed nature of the algorithm. Dependent on type of artifact and data to be cleaned, however, a combination of approaches employing specific detection metrics might be needed in order to reduce unwanted signals to an acceptable level. It is noteworthy to know that validation has so far been performed in healthy volunteers only, making it difficult to judge when fully automated methods will meet clinical standards.

### 4.2. Head Movement

Head or more general subject movement during signal acquisition can significantly affect the usability of MEG data due nonneuronal signals arising from muscle activity, artifacts caused by motion related scanner vibrations and changes in spatial reference impacting subsequent source localization. Typically, healthy adult patients are highly motivated and maintain a constant head position within about 2 mm standard deviation of the measured head positions, in particular when scans are short. Movement related artifacts, however, might increase substantially in certain patient populations and in pediatric measurements [4]. Early approaches aimed at preventing head movement as far as possible with deflatable support cushions, bite-bars, or other constraints. In contrast, modern approaches increasing rely on continuous position monitoring in conjunction with computational methods to correct for head movements during data preprocessing.

Arguably, the most advanced approaches utilize signal-space-separation (SSS). This technique is based on fundamental properties of quasi-static magnetic fields implying that the measurement geometry can be divided into two source volumes with respect to the sensor array. The internal volume is a sphere whose radius is defined as the distance from the head origin to the nearest MEG sensor. The external volume is defined as the outside of a sphere where the radius is the distance from the head origin to the farthest MEG sensor. Then, the magnetic field measured by the sensors (*M*) located in the current-free space between the internal and external volumes can be represented as

(8) M≔Mint⁡+Mext=H×A=[Hint⁡Hext][Aint⁡Aext]  =Hint⁡×Aint⁡+Hext×Aext,

where *H* depends on the sensor array and its location with respect to the two source volumes. In contrast, the multipole moments *A* are device and geometry independent and can be estimated from the original measurement *M*. Only the *internal* terms in the formula above are of interest as they are assumed to represent sources in the brain, and a signal-space separated (filtered) signal is obtained by omitting the *external* terms, thereby suppressing artifacts originating far away from the MEG sensors. Critically, the device independency of *A* allows correction of head movements according to the following:

(9) $$\begin{array}{c} M_{s}=H_{s}\times A_{\mathrm{int}}, \end{array}$$

where *H*_*s*_ represents a virtual sensor array locked to the subject's head. In other words, one can calculate the signals recorded by a sensor array with respect to where the head is stationary, provided that a continuous movement monitoring method is available [71, 72]. A temporal extension of this approach, known as tSSS, attempts to identify residual signals common to *M*_int⁡_ and *M*_ext_. Such signals typically but not necessarily represent artifacts originating between the inside and outside volumes and, once identified, can be removed from *M*_int⁡_ using a projection as described previously [73].

The SSS approach has been tested extensively, where the suppression of artifacts originating at a large distance (>1 m) from the scanner matches the theory-based expectation [74, 75]. Note that suppression of nearfields such as ocular and cardiac artifacts can be unsatisfactory. Excellent results, however, have been obtained for tSSS in the case of speech artifacts, where the movements of tongue, lips, and jaw, and the activity of facial muscles produce magnetic fields interfering with the signals [76]. This appears to hold also for magnetic fillings and other dental implants, significantly increasing the number of cases that can be successfully scanned with MEG.

Although movement correction based on SSS has been validated using artificial sources, relatively few studies have investigated the effects of head motion in vivo. Initially, the location error of the early somatosensory response to electrical median nerve stimulation was calculated in a single subject making controlled head movements [77]. The authors reported a 3-fold reduction in localization error compared to raw data when using tSSS-based movement correction for head shifts up to 3 cm. The limit of 3 cm is broadly consistent with a single-case study reporting consistent localization of epileptic activity when correcting for head movement during seizure (2.3 arc length), whereas activity located in different sublobar regions without correction [78]. (Note the MEG results did not change patient management; see Section 5.1 for a discussion of MEG in epilepsy research.)

A recent study systematically investigated the effects of head motion in a larger sample size [79]. The authors recorded the neuronal response to auditory and somatosensory stimuli in 20 healthy volunteers performing controlled head movements. Moreover, magnetized particles were attached to the subject's head to simulate nearby interference sources, and a phantom head containing current dipoles was used for cross validation. Confirming and extending previous findings, the results showed that source locations can be recovered reliably with SSS (error about 5 mm) for head shifts not exceeding 3 cm. Also, using tSSS to remove artifacts due to nearby sources did not affect the reference data. The authors pointed out that auditory evoked responses appeared to be more affected by continuous movement than somatosensory responses, implying that the outcomes of motion correction have to be judged in a context dependent manner.

### 4.3. Implanted Materials

Implanted, metal containing objects such as electrodes, hydrocephalus shunts, or ferromagnetic remnants of neurosurgical operations pose particularly high challenges for MEG artifact correction, as non-physiological magnetic fields are generated “in situ” directly interfering with the brain signals. The artifacts become even worse when electrically active devices are involved, such as brain stimulators or pacemakers. There is currently no universally applicable method available that corrects for these types of artifacts, however, promising results are beginning to emerge. Two examples may suffice for illustration here.

One study used MEG to record epileptic discharges in patients carrying a vagus nerve stimulator, which has proven beneficial to some epileptic patients considered unsuitable candidates for resective surgery [80]. Using tSSS to clean the data, the authors were able to obtain interpretable signals in 7 out of 10 patients. The clean signals were subsequently modeled using equivalent dipoles in order to localize interictal spikes. As a consequence of the MEG results, management changed to invasive video-EEG (IVEM) in two patients. Furthermore, the MEG findings supported the proposed management in four patients, demonstrating the feasibility of MEG in patients with a nerve stimulator.

Another study recorded simultaneous MEG and local field potentials in 17 subjects with Parkinson's disease (PD) carrying a deep brain stimulator (DBS) to alleviate symptoms caused by degrading motor control [81]. Using beamforming, the authors were able to suppress the high-amplitude artifacts caused by the DBS wire and electrode and extract interpretable virtual electrode time-series. Subsequent time-frequency analysis pointed to specific synchronization in the gamma band (60–90 Hz) that stimulates movement through modulatory effects in the basal ganglia cortical network. Although not of direct clinical relevance, this finding is of importance for a better understanding of motor control in PD.

## 5. Clinical Application: Epilepsy

The most well-established clinical application of MEG is presurgical evaluation in epilepsy. Epilepsy is a diverse set of neurological disorders characterized by the tendency to have recurring seizures. A seizure is the response to an abnormal electrical discharge in the brain, and it describes a variety of behaviors, such as psychic aberrations (e.g., a sense of déjà vu), abnormal sensations (e.g., sensing an intense smell), impairments in speech or speech comprehensions, and coordinated and involuntary movements. Both the nature and severity of seizure symptoms are dependent on which parts of the brain are affected by the abnormal discharges. Seizures of low severity leave consciousness unimpaired, whereas the most severe seizures may become life-threatening and require instant medical attention [82, 83]. 

Often it is unknown what causes abnormal electrical discharges; however, certain risk factors have been identified, such as brain lesions, disorders of the metabolic system, brain infections, and drug and substance abuse amongst others. A seizure typically lasts up to several minutes implying that the time period that exists between seizures (interictal period) accounts for the majority of a patient's life with epilepsy. Although most patients may appear symptom free during interictal periods, abnormal discharges may be present within the brain. The anatomic origin of such discharges is often, although not always, close to the anatomic origin of seizure-related (ictal) discharges, implying a clinical relevance of interictal epileptic activity [84].

In industrialized countries, epilepsy has an estimated prevalence of 4–10 per 1000 and an incidence of 40–70 per 100,000 and year causing substantial medical expenditures. The disorder is usually controlled, but not cured, with antiepileptic drugs, resulting in seizure freedom in about 60% of patients. The remaining 40% of affected individuals, however, have to be considered pharmacoresistant [85]. Surgery is an alternative option in these cases if the seizures can be traced to abnormal electrical discharges in one or more clearly delineated brain areas. Then, the removal or disconnection of the epileptogenic tissues can entail high rates of success. Epilepsy surgery, however, faces substantial challenges. The ultimate goal is improvement of quality of life while avoiding cognitive or other impairments caused by damage to essential functional areas. This requires a substantial, multitechnological, and possibly invasive pre-operative workup aimed at both the accurate localization of the epileptic foci and mapping of nearby essential functional areas, where only the combined results can enable successful surgery [86].

### 5.1. Localization of Epileptic Foci

**Figure 3:**
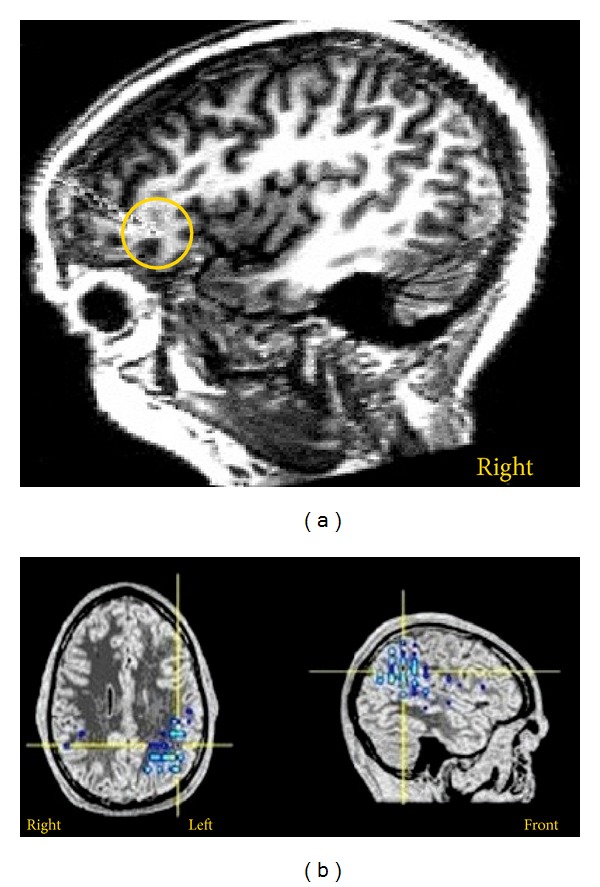
MEG in presurgical evaluation. (a) Equivalent current dipole localization (center of circle) for an interictal discharge in a patient with right frontal epilepsy. (b) Distributed source modeling of language function in left hemisphere Wernicke's area for a verb generation task (adapted from [93]).

The potential of MEG to localize epileptogenic regions was communicated to the wider scientific audience over 25 years ago [87]. Efforts at confirming the accuracy of MEG source localizations have been addressed from various indirect and direct approaches. Indirect validation comes predominantly from studies showing colocalization of the magnetic source estimates with known epileptogenic tissues apparent on structural and functional MR imaging, or confirmed with invasive intracranial EEG (IC-EEG) and successful surgical outcomes. In contrast, the direct methods reflect studies utilizing simultaneous MEG and IC-EEG recordings, or simulated epileptic discharges generated by implanted dipoles [85, 88]. Similar to other non-invasive approaches to preoperative evaluation, a considerable amount of the MEG work has been based on interictal discharges (an example is shown in Figure 3(a); [55, 89–91]). However, the importance to determine the location of the onset of ictal activity associated with clinical seizures has prompted researcher to address methodological challenges and validate ictal MEG [92].

In one of the first larger studies of its kind, successful ictal MEG recordings were made in 6 out of 20 patients with intractable complex partial seizures [94]. All patients had neocortical epilepsies, and data collection took place over a span of 3 years. In order to capture ictal-onset activity, a physician remained in the magnetically shielded room with each patient and retracted the probe soon after a seizure occurred, allowing the patient to move freely. During recording, a second physician monitored real-time displays of the MEG and EEG waveforms and triggered data collection when abnormal discharges were detected. On average, the ictal scans took several hours, and the patients were allowed light sleep unless it was necessary to reposition the head, or if sleep to wake transitions were felt necessary to obtain ictal data. This allowed the authors to record a few seconds of ictal data, although their procedure had a limited capability to track seizure spread.

The authors used single equivalent current dipole to localize sources underlying the MEG field patterns detected during ictal and interictal data segments. Compared to IC-EEG, the authors reported that ictal MEG provided localizing information that was superior to interictal MEG in three of the six patients. Localization of ictal onset by MEG was found to be equivalent to invasive EEG in five of the six patients. In one patient, ictal-intracranial EEG showed nonlocalizing diffuse seizure related activity, whereas the MEG results pointed to a circumscribed ictal onset zone in right prefrontal cortices. A resection was performed, based in part on the MEG findings, leaving the patient seizure free and without postoperative neurocognitive deficit over 2 years of followup. Despite a small number of ictal recordings, the authors rightly concluded that ictal MEG might be superior to invasive recordings under certain conditions.

The study furthered two previous investigations in 4 patients [95] and 3 patients [92] with epilepsy, respectively. Both studies concluded that the ictal MEG results were in agreement with invasive and noninvasive preoperative assessments. Also the ictal and interictal MEG source localizations were topographically very similar. These findings, however, were rather preliminary in nature and insufficient information was available as to assess the reliability of the utility of MEG in identification of epileptogenic zones.

Further support for the role of ictal MEG in presurgical evaluation is provided by a recent study in children with intractable complex partial epilepsy [96]. Over a span of 3.5 years, the authors successfully obtained ictal data in 8 pediatric patients who subsequently underwent surgical resection. During MEG recordings, the patients were either sedated or spontaneously attained stage II sleep because of partial sleep deprivation the night before the study. The authors used single ECD modeling to estimate ictal sources based on high-pass filtered MEG data, where the individual frequency cutoff was determined with short-time Fourier transform. The patient head position was continuously monitored, and only recordings with less than 5 mm head movement were accepted. The concordance between the MEG source localizations and IC-EEG was evaluated at three levels of anatomical resolution as follows: hemispheric lateralization, lobar localization, and sublobar localization (e.g., mesial versus lateral).

The authors observed that 5 out of 8 patients had concordant interictal discharge and ictal onset MEG localizations in the same lobe; however, the ictal source was in general closer to the seizure-onset zone as determined by intracranial EEG. The authors also reported a high correlation between MEG source localization and surgical outcome, in particular when the ictal and interictal MEG source localizations showed agreement with respect to lateralization and lobar localization, and only one type of seizure semiology was present. Acknowledging that the capture of seizures during MEG recording was challenging, the authors concluded that ictal MEG onset can provide useful additional information for surgical evaluation of patients with intractable epilepsy.

This study is interesting in that the authors complemented their ECD calculation, which is currently the only clinically accepted method, with other source estimations, such as minimum norm and beamforming techniques. The findings suggest that these methods can yield independent information about the size of the initial ictal-onset zone and its subsequent propagation. However, the data were too preliminary in order to reach firm conclusions.

Most recently, a study reviewed the MEG data of well over 200 epilepsy patients that had undergone various stages of clinical assessment and treatment over a total period of 14 years [97]. The authors identified 12 cases who had surgery and presented sufficient data to allow retrospective comparison of interictal and ictal MEG with intracranial EEG. Spatialtemporal signal separation was used to control artifacts caused by seizure-related head movements. Epileptic discharges were modeled using an iterative scheme yielding a cluster of single equivalent dipoles that best explained the seizure-related activity. The resultant ECD clusters were classified according to two models of different anatomic resolutions in order to compare the source location of epileptic activity recorded by MEG and intracranial EEG. The authors' high-resolution hemisphere-lobe-surface model was based on hemisphere, lobe, and surface of the lobe. The low-resolution hemisphere-lobe model was based on hemisphere and lobe only. 

Taking intracranial EEG as the standard for estimating ictal-onset zones, the authors reported high sensitivity (96%) and high specificity (90%) of ictal MEG at low resolution. At high spatial resolution, a lower sensitivity (71%) and specificity (73%) of ictal MEG were observed, where the specificity was about equal to interictal MEG (77%). In contrast, the sensitivity of interictal MEG was low (40%). None of their operated patients had mesial temporal lobe epilepsy; however, the authors found that ictal MEG was equally sensitive and specific on dorsolateral and nondorsolateral neocortical surfaces up to a depth of 4 cm. Despite robust data, the authors were rather moderate in concluding that ictal MEG might have some advantages over interictal recordings regarding the sensitivity with which epileptic foci are detected compared to invasive approaches.

This study further demonstrates the utility of MEG in evaluation of patients with epilepsy. However, important issues remain. Three patients, out of a larger pool of 22, who underwent surgery reached Engel classes I (free of disabling seizures) to III (worthwhile improvement) but had no ictal MEG signal suggesting that MEG alone might not be sensitive enough on its own in some cases. Challengingly, ictal onset is a highly complex dynamic process, where different types of MEG waveforms in different frequency bands appear after the initial spike. This poses important questions for future investigations as to what are the clinical significance of different MEG waveform types, and which source modeling approaches are most appropriate to extract clinical information.

To this end, challenges will continue to exist. Epilepsy simply is a highly complex disorder implying intricate changes in the neuronal dynamics, where it is possible that neither IC-EEG nor surgical seizure-free outcome reliably reflects epilepsy localizations that define brain areas responsible for the patient's seizures [85]. Notwithstanding such fundamental issues, MEG has been recognized as a tool in epilepsy diagnostic mainly as part of the first phase of presurgical evaluation complementing structural MRI, combined video/surface-EEG monitoring, and, in some cases, SPECT and PET [98]. In particular, there is growing evidence that MEG can, at least in some cases, identify epileptogenic lesions not visible in structural scans [99]. It is worth noting that MEG is not a replacement for surface EEG as these technologies are complementary with respect to sensitivity to epileptic discharges, that is, either method can detect spikes not seen by the other. It is not fully resolved what causes these differences; however, the relative insensitivity of MEG to deeper, radial sources (as in temporal lobe epilepsies) paired with a better signal-to-noise ratio for sources in superficial, neocortical areas is likely to be explanatory factors [100–102].

### 5.2. Lateralization of Language

Deterioration or even loss of language function is considered a dramatic cognitive complication of surgical removal of brain tissue performed to alleviate otherwise intractable neuropathological conditions. Therefore, accuracy in the lateralization and localization of language function is of great importance to the clinician when considering ablation of tissue. The intracarotid amobarbital procedure (IAP; also known as Wada test) has been the gold standard for lateralization of language dominance before surgery since its introduction more than 60 years ago. It is based on injection of amobarbital separately into the left and right carotid arteries to deactivate one cerebral hemisphere at a time. Concomitantly, the language functions of the nonanesthetized hemisphere are tested with a variety of simple tasks (e.g., the patient is asked to follow simple commands, name objects, or count the days of the week). If one hemisphere is language dominant, significant language disturbances are observed with injection on only that side. Usually, the IAP is preceded by an angiogram in order to map the cerebral vascular structure, forecast how the anesthetic will be distributed in the hemispheres, and detect possible contraindications precluding the IAP [103, 104].

Despite its clinical success, however, the IAP is an invasive procedure involving substantial risk of serious complications due to the angiogram testing and subsequent anesthesia of the brain. Moreover, it is known that the test can pose difficulties in interpretation of results because of, amongst others, crossflow of the anesthetic to the other hemisphere, insufficient anesthesia of all language areas, interactions between amobarbital and certain antiepileptic drugs, and possible dissociation of language functions within an individual, where specific functions are represented in each hemisphere. Seeking alternative techniques, however, is not an easy task because of the nature of the comparison. The IAP relies on language testing during a transient inactivation of one hemisphere, whereas electrophysiology or metabolism-based approaches assess the spatial and temporal patterns of brain activity during language-related tasks [105].

One of the largest studies of the concordance of language dominance between MEG and IAP was conducted in 85 patients with intractable seizures evaluated for epilepsy surgery [106]. The subjects were required to recognize spoken words and determine whether each word had been in a list of target words presented before testing. Such test of verbal memory elicits specific event-related fields at long latency (200–1000 m after stimulus onset) thought to reflect neuronal activity underlying the execution of higher cognitive functions such as recognition judgments, syntax, and semantic processing [107]. Using single equivalent dipoles models, the authors observed neuronal sources in the posterior portion of the superior temporal gyrus in all patients. Secondary sources were found in inferior frontal, middle, and mesial temporal regions to varying degree across subjects. Interestingly, these secondary areas were often found bilaterally, even for those patients who were unilateral language dominant according to the IAP. The authors calculated a laterality index based on clusters of dipoles for each subject as follows:

(10) $$\begin{array}{c} \text{LI}=\frac{N_{R}-N_{L}}{N_{R}+N_{L}}, \end{array}$$

where *N*_*R*_ and *N*_*L*_ denote the number of dipole clusters in the right and left perisylvian regions, respectively. A laterality index between −0.1 and 0.1 was considered to indicate bilateral symmetric activation whereas indices smaller than −0.1 or greater than 0.1 indicated left or right hemisphere dominance. Using this index, the authors found a strong concordance between the MEG and IAP results (87%).

In the majority of the discordant cases, the dipole analysis suggested bilateral language whereas the IAP showed left hemisphere dominance (64%). In order to further assess the clinical usefulness of the MEG approach, the author performed a separate, albeit related analysis by assessing the potential presence of eloquent cortex in the hemisphere of seizure onset. The concordance between MEG and IAP results was high, where both methods determined either the presence or absence of language function in the affected hemisphere in 93% of patients (98% sensitivity, 83% selectivity). An analysis of the discordant cases suggested that MEG provided a more conservative test for the presence of language function in the hemisphere subject to surgery, where 6% of patients presented language function in the affected side according to the MEG but not IAP.

The same experimental approach was used in a replication study performed several years later [108]. Based on a smaller sample of 35 patients with epilepsy and/or brain tumor undergoing presurgical evaluation, the authors reported a concordance of 69% between the MEG and API results regarding hemisphere language dominance. Regarding the presence or absence of language function in the affected hemisphere, the concordance between the MEG and IAP results was comparable to the original study (86%; 80% sensitivity, 100% selectivity). An analysis of the discordant cases, however, suggested that the IAP provided a more conservative test for the presence of language function in the hemisphere subject to surgery, where all discordant cases had language function in the affected side according to the IAP but not the MEG. The authors argued that their results are broadly consistent with the original study, where an unusually high rate of atypical IAP language cases in this sample were believed to explain the noted discrepancies.

An alternative experimental approach to assess language dominance with MEG was recently employed, where 63 patients with intractable epilepsy, brain tumors, or vascular lesions were asked to silently read visually presented words [109]. The authors used beamforming to extract source activation curves at individual voxels (virtual electrodes) from the sensor data and calculated event-related desynchronization for the *β* (13–25 Hz) and low *γ* (25–50 Hz) frequency bands. Using a laterality index for their ERD measure, the authors reported a concordance between the MEG and IAP of 81%. The authors argued that functional imaging with spatially filtered MEG based on oscillatory changes was competitive with conventional ECD approaches with respect to estimating language dominance. Interestingly, the authors achieved a high concordance based on activity in frontal language areas, suggesting ERD can probe distributed language systems beyond posterior areas that are commonly modeled with ECD-based methods [110–112].

These results have been corroborated by a number of studies, typically based on smaller sample size, investigating hemispheric language lateralization and localization of language within hemispheres (an example is shown in Figure 3(b)). Reliability and concordance of results hold for a variety of task as such as word recognition, word categorization, and semantic judgments with both auditory and visual stimuli (for a review see [113]). Taken the evidence together, one can safely argue that MEG is highly reliable relative to the IAP in the determination of language dominance and is able to provide robust intrahemispheric localization of language. As such the MEG is comparable to fMRI, the prime non-invasive tool for presurgical language evaluation. 

Currently, fMRI is more widespread than MEG and has some advantages due to higher standardization of technology, protocols, and analytical methods [103]. In contrast, MEG scanning is absolutely risk-free, entails the least discomfort, and might be faster than fMRI for certain protocols. Moreover, there is some recent evidence pointing toward fMRI-based language lateralization being sensitive to cerebral lesions and to problems in interpreting activation patterns in patients with atypical language representation [114]. The MEG might be more robust in these respects, although further studies are needed to understand the effect of lesions and other factors on localization of language function in order to minimize the risk of physicians and scientists misinterpreting results.

## 6. Clinical Research: Autism

Autism is a heterogeneous neurodevelopmental disorder that is associated with a triad of characteristic symptoms: first, impairments in social interaction manifested by failure to develop appropriate peer relationships, lack of share of enjoyment, lack of social and emotional reciprocity, or impairment in the use of multiple nonverbal behavior; second, impairments in communication as manifested by delay in the development of spoken language, impairment in the ability to initiate or sustain a conversation, or lack of spontaneous appropriate social play; third, restricted, repetitive, and stereotyped patterns of behavior, interests, and activities as manifested by inflexible adherence to specific nonfunctional routines or rituals, stereotyped and repetitive motor mannerisms (e.g., hand or finger flapping), or persistent preoccupation with objects. Autism is one of the recognized disorders in the autism spectrum, ASD for short [115, 116].

The disorder becomes apparent within the first three years of life and continues throughout adolescence and adulthood, although the manifestations of the condition change over time and there is marked interindividual phenotypic variability. The diagnosis of ASD is based solely on clinical assessment of behavior [117]. There is no known cure and there is no apparent unifying mechanism that may underlie ASD at the genetic, molecular, cellular, or systems level [118, 119]. The prevalence of ASD is currently estimated at 6 in 1000. On average, males are at higher risk for ASD than females by a ratio of about 4 : 1. Comorbidity with genetic disorders such as tuberous sclerosis and Fragile X can occur, and up to a third of individuals with autism suffer from epilepsy [120].

### 6.1. Epileptiform Activity in ASD

An important issue in developmental neurosciences is the contribution of epilepsy to autism spectrum and acquired language disorders. This importance derives from the observation that children with Landau-Kleffner syndrome (LKS), ASD, or developmental language disorders, who show common impairments in generating and decoding speech, are at higher risk for epilepsy than children with the fluent, albeit typically aberrant, language of verbal children with autism [121–123]. Despite considerable research into this issue, there appears to be only two MEG studies investigating epileptic brain activity in individuals with ASD.

Initially, MEG was used to record the electrophysiological response during stage III sleep in 50 children with regressive ASD with onset between 20 and 36 months of age (15 out of 50 with a clinical seizure disorder) and 6 children with LKS [124]. All children, with or without clinically relevant seizures, occasionally demonstrated unusual behaviors (e.g., rapid blinking, unprovoked crying, and brief staring spells) which, if found in a normal child, might conceivably be interpreted as indicative of subclinical epilepsy. Using equivalent current dipoles to model neuronal sources, the authors found epileptic brain activity in all children with LKS (5 had complex seizures), where generators are located predominantly in left intra- and perisylvian regions.

MEG identified abnormal discharges in 82% of the children with ASD. When epileptic activity was present in the ASD, the same sylvian regions seen in LKS were active in 85% of the cases. Moreover, 75% of the ASD children with epileptic discharges demonstrated additional nonsylvian zones. Interestingly, simultaneous EEG revealed epileptic activity in only 68% of the children with ASD. Despite the multifocal nature of the epileptic discharges, neurosurgical intervention aimed at control led to a reduction of autistic features and improvement in language skills in 12 of 18 cases.

It is generally agreed that this study has provided some evidence that there is a subset of individuals with ASDs who demonstrate epileptic activity that might be associated with autistic symptoms, even in the absence of a clinical seizure disorder. Also, MEG appeared to have significantly greater sensitivity to this epileptic activity than simultaneous EEG, reinforcing the role MEG plays as a clinical tool. The conclusions drawn from this study, however, have been criticized on grounds of referral bias, methodological shortcomings, and possibly conflating regressive autism and LKS [121, 125].

Several years later, MEG was used to study spontaneous neural activity in 36 children with autism spectrum disorder [126]. The patients were referred for full evaluation and workup studies, although none of the subjects had a diagnosis of seizure disorders. A visual inspection of the MEG data by a trained examiner blinded to the subject's clinical history revealed specific abnormalities in the form of low amplitude monophasic and biphasic spikes as well as acute waves in about 86% of all children with ASD. Source estimates based on equivalent current dipole modeling suggested generators predominately in perisylvian regions. Notably, epileptic spikes were mostly found in the right but not left hemisphere in individuals with Asperger's syndrome (a subtype within ASD). In contrast, simultaneous EEG recordings revealed abnormal activity in only 3% of the cases.

This study provides further evidence that subclinical epilepsy can be detected in many children with ASD using MEG which appeared to have greater sensitivity than EEG in this study. Moreover, there was some evidence suggesting that clinical subtypes within ASD might be distinguishable according to laterality of abnormal discharges. Further conclusions, however, could not be drawn, as comparing typically developing children were not investigated.

Note that a recent study used single-photon emission computed tomography (SPECT), EEG, and MEG to investigate ictal and interictal discharges in 15 individuals with a diagnosis of ASD and seizure disorder [127]. Somewhat unusual, the authors did not report their MEG results. However, the EEG and SPECT localizations of epileptic foci appeared concordant. Interestingly, the authors found no significant relationship between the regions of hypoperfusion as measured by SPECT and autism symptom severity. This might imply that epilepsy is not a causal factor for ASD. Rather epileptic discharges simply occur in areas of the brain that are also functionally or pathologically involved in ASD.

### 6.2. Face Processing

It is well documented that individuals with ASD show impairments that are face linked, for example, in patterns of eye contact and response to gaze. During development, individuals with ASD typically show reduced memory for faces and a deficit in recognizing facially expressed emotion [128]. Autism, however, is also associated with a heightened ability to recognise upside down faces and to extract information from some face features compared to typically developing individuals. Such observations support the suggestion that individuals with autism attend to individual features rather than process faces as a whole [129, 130]. Most of the neuroimaging literature on face processing in autism has concentrated on the fusiform face area (FFA) located in ventral occipitotemporal cortices. The FFA is part of the brain's face processing system and is selectively activated by images of faces in typically developing subjects. It is generally accepted that FFA activation is atypical in individuals with ASD [131, 132]. However, there is debate as to whether this is intrinsic or is connected with the details of the task, such as the degree of engagement. Despite the relevance of face processing to the study of autism, there have been only a few published MEG studies in individuals with ASD so far.

**Figure 4:**
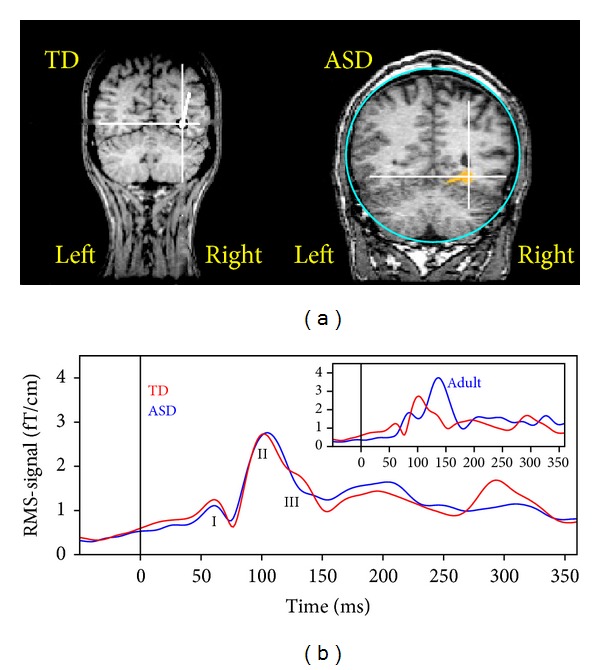
MEG in autism research. (a) The ECD locations for responses to images of human faces at about 145 ms after stimulus onset in a typically developing subject and an individual with ASD (circle indicates the volume conductor sphere). These images illustrate locations in the right posterior cortices of the generators, where, on average, dipole locations are more lateral in TD compared to ASD. (b) Grand root-mean-square signals following face images. The curves have been obtained by summation over all participants within a participant group (blue, boys with ASD; red, typically developing boys; and stimulus onset at 0) and channels. Even in middle childhood, the neural mechanisms underlying face processing are less specialized than in adults (inset) with greater early activation of posterior occipital cortices (I, II) and less specific activation of ventral occipitotemporal cortex (III), particularly in boys with ASD.

MEG was used to study the neuronal response in 12 able adults with ASD and 22 adult controls performing image categorization and 1-back image memory task [7]. The authors observed that the response to images of faces in right FFA at about 145 ms after stimulus onset was significantly weaker, less lateralized (Figure 4(a)), and less affected by memory recall in patients compared to typically developing subjects. Moreover, an early latency (30–60 ms) response to face images over right anterior temporal regions was observed during memory encoding but not memory recall in control subjects, whereas activity was observed during recall but not encoding in patients. The authors argued that their individuals with ASD might have developed differently located and functionally different extrastriate processing pathways. These pathways seem functionally capable of at least some aspects of face processing. It is unresolved, however, how such processing routes relate altered socially linked cognition.

Subsequently, MEG was used to study face processing in 10 children with ASD and 10 mental age and gender matched children who were typically developing boys aged 7–12 years [8]. The authors reported that the response differences between the two groups of children were less marked than between the adult groups and between children and adults. There were subtle differences, however, with greater recruitment of occipito-temporal cortices in processing nonface stimuli and a less face specific response in children with ASD compared to controls. Based on these findings, the authors hypothesized that the neural mechanisms underlying face processing are less specialized in children than in adults even in middle childhood, where there appears a divergence between the normal and abnormal developing face and object processing systems (Figure 4(b)).

Most recently, MEG was used to record the response to Mooney stimuli in 13 adult individuals with ASD and 16 healthy controls matched for age, gender, and IQ [133]. Mooney images consist of degraded pictures of human faces where all shades of gray have been removed, leaving the highlights in white and the shadows rendered in black. Most typically developing subjects perceive a face when presented in a Mooney image, but fail to do so when the image is inverted [134]. The authors observed that individuals with ASD showed reduced detection rates during the perception of upright Mooney stimuli, while responses to inverted images were in the normal range. Concerning the electrophysiological level, the authors reported that perception of faces involved increased high gamma (60–120 Hz) activity in a fronto-parietal network in typically developing subjects, whereas in the ASD group stronger activation was observed in posterior visual regions. The authors have argued that their data provide novel evidence for the role of gamma-band activity in visuoperceptual dysfunctions possibly underlying impairments in face processing.

### 6.3. Summary: Autism

Beginning with the late 1990s, MEG has been used increasingly as an investigative tool to study the neurophysiological basis of autism spectrum disorders, mirroring a general trend of intensified usage of neuroimaging technologies in developmental disorders and pediatrics [3]. Over and above epilepsy and face processing, research has been directed at characterizing and understanding various aspects of neural processing which are affected amongst individuals on the autism spectrum, such as auditory processing [135], semantic processing [136], and theory-of-mind progressed through study of imitation-related processes [137]. The present MEG contributions to autism research, however, are still limited in that studies are rather fragmented in terms of experimental design and scientific objectives, sample sizes are often typically low, subject matching criteria vary substantially between investigations, and comparisons are often limited to ASD and typically developing populations rather than pathologies exhibiting some overlap with the autistic spectrum. Nonetheless, MEG research into autism has provided insight in line with the more general observation that affected individuals may use different strategies to accomplish familiar tasks [129, 138].

## 7. Conclusion

Since its introduction a little over 40 years ago, magnetoencephalography has come a long way being now regarded as one of the most modern imaging tools available to radiologists. Many institutions, laboratories, and hospitals all over the world are increasingly embracing this noninvasive technology. However, the role of MEG in diagnosis, prognosis, and patient treatment is still limited and MEG research applications will have to mature before they can become clinically relevant. Moreover, there are challenges inherent in the technology that will have to be addressed in the near future. MEG is relatively expensive, it is reliant on natural gas resources for normal operation, and analytical approaches have not been standardized yet. Despite its disadvantages, MEG is an exquisitely patient friendly tool capable of contributing indispensable information about brain dynamics not easily obtained with other technologies. Ultimately, any neuroimaging technology has inherent limitations, and it is likely that a combination of several modalities including MEG will unveil the most information about the neuronal correlates of behavior under normal and pathological conditions. Thus, it appears that MEG is beginning to fulfill the potential as a clinical tool for a wide range of disorders.

## Figures and Tables

**Table 1:** Comparison of distributed source algorithms often used in clinical MEG research.

Name	*p*	*W*_model_	Comment
MNE	2	$\underline{1}$	+ simple − currents packed towards surface of volume
MNE (weighted)	2	$\underline{1}$/current-gain	+ no surface bias − blurry images
LORETA	2	Laplacian^+^	+ deep source accurate − estimates superficial sources too deep
MCE	1	$\underline{1}$/current-gain	+ focal estimates − strong sensitivity to noise

MNE: minimum norm estimate (e.g., [42]). MCE: minimum current estimate [46]. LORETA: low resolution brain electromagnetic tomography [45]. ^+^Mathematical operator representing flux density and gradient flow of a function.

**Table 2:** Overview of some of the relevant ICA-based methods for ocular and/or cardiac artifact removal in chronological order.

First author	Artifact	Electrode	Sample	Comment
Rong [65]	Ocular, cardiac	No	5	One of the first automated removal methods; requires a predetermined artifact template
Okada [66]	Ocular	Yes	7	Reliable performance for blinks occurring in rapid succession
Mantini [67]	Ocular, cardiac	No	12	Requires complex parameter tuning; can correct environmental artifacts
Dammers [68]	Ocular, cardiac	Yes	6	Uses signal amplitudes and phases; can correct for muscle artifacts
Klados [69]	Ocular	Yes	27	Hybrid regression-ICA approach; available as open source

Electrode: indicates whether an electrooculogram and/or electrocardiogram measurement is required. Sample: number of healthy volunteers used for validation.

## References

[1] Cohen D (1972). Magnetoencephalography: detection of the brain’s electrical activity with a superconducting magnetometer. *Science*.

[2] Pataraia E, Baumgartner C, Lindinger G, Deecke L (2002). Magnetoencephalography in presurgical epilepsy evaluation. *Neurosurgical Review*.

[3] Paetau R (2002). Magnetoencephalography in pediatric neuroimaging. *Developmental Science*.

[4] Mäkelä JP, Forss N, Jääskeläinen J, Kirveskari E, Korvenoja A, Paetau R (2006). Magnetoencephalography in neurosurgery. *Neurosurgery*.

[5] McDonald CR (2008). The use of neuroimaging to study behavior in patients with epilepsy. *Epilepsy and Behavior*.

[6] Schwartz ES, Edgar JC, Gaetz WC, Roberts TPL (2010). Magnetoencephalography. *Pediatric Radiology*.

[7] Bailey AJ, Braeutigam S, Jousmäki V, Swithenby SJ (2005). Abnormal activation of face processing systems at early and intermediate latency in individuals with autism spectrum disorder: a magnetoencephalographic study. *European Journal of Neuroscience*.

[8] Kylliäinen A, Braeutigam S, Hietanen JK, Swithenby SJ, Bailey AJ (2006). Face- and gaze-sensitive neural responses in children with autism: a magnetoencephalographic study. *European Journal of Neuroscience*.

[9] Dima D, Frangou S, Burge L, Braeutigam S, James AC (2012). Abnormal intrinsic and extrinsic connectivity within the magnetic mismatch negativity brain network in schizophrenia: a preliminary study. *Schizophrenia Research*.

[10] Nakamura M, Watanabe S, Inagaki M (2013). Electrophysiological study of face inversion effects in Williams syndrome. *Brain and Development*.

[11] Paetau R (2009). Magnetoencephalography in landau-kleffner syndrome. *Epilepsia*.

[12] de Haan W, van der Flier WM, Koene T, Smits LL, Scheltens P, Stam CJ (2012). Disrupted modular brain dynamics reflect cognitive dysfunction in Alzheimer’s disease. *NeuroImage*.

[13] Cheng C, Wang P, Hsu W, Lin Y (2012). Inadequate inhibition of redundant auditory inputs in Alzheimer’s disease: an MEG study. *Biological Psychology*.

[14] Takei Y, Kumano S, Hattori S (2009). Preattentive dysfunction in major depression: a magnetoencephalography study using auditory mismatch negativity. *Psychophysiology*.

[15] Dockstader C, Gaetz W, Cheyne D, Tannock R (2009). Abnormal neural reactivity to unpredictable sensory events in attention-deficit/hyperactivity disorder. *Biological Psychiatry*.

[16] Helenius P, Laasonen M, Hokkanen L, Paetau R, Niemivirta M (2011). Impaired engagement of the ventral attentional pathway in ADHD. *Neuropsychologia*.

[17] Salmelin R (2007). Clinical neurophysiology of language: the MEG approach. *Clinical Neurophysiology*.

[18] Laaksonen K, Helle L, Parkkonen L (2013). Alterations in spontaneous brain oscillations during stroke recovery. *PLoS ONE*.

[19] Lewine JD, Davis JT, Bigler ED (2007). Objective documentation of traumatic brain injury subsequent to mild head trauma: multimodal brain imaging with MEG, SPECT, and MRI. *Journal of Head Trauma Rehabilitation*.

[20] Franzen JD, Wilson TW (2012). Amphetamines modulate prefrontal gamma oscillations during attention processing. *Neuroreport*.

[21] Hämäläinen M, Hari R, Ilmoniemi RJ, Knuutila J, Lounasmaa OV (1993). Magnetoencephalography—theory, instrumentation, and applications to noninvasive studies of the working human brain. *Reviews of Modern Physics*.

[22] Hari R, Forss N (1999). Magnetoencephalography in the study of human somatosensory cortical processing. *Philosophical Transactions of the Royal Society B*.

[23] Vrba J (1999). Multichannel squid biomagnetic systems. *Applications of Superconductivity*.

[24] Vrba J, Robinson SE (2001). Signal processing in magnetoencephalography. *Methods*.

[25] Vrba J (2002). Magnetoencephalography: the art of finding a needle in a haystack. *Physica C*.

[26] Plonsey R (1982). The nature of sources of bioelectric and biomagnetic fields. *Biophysical Journal*.

[27] Wu J, Okada YC (1998). Genesis of MEG signals. II. Effects of manipulating voltage- and Ca(2+)-activated K(+) channels on the magnetic fields produced by the CA3 slice of guinea pig. *Recent Advances in Human Neurophysiology*.

[28] Wu J, Okada YC (1998). Genesis of MEG signals. I. Effects of ligand-gated channels on the magnetic fields produced by the CA3 slice of guinea pig. *Recent Advances in Human Neurophysiology*.

[29] Hansen PC, Kringelbach ML, Salmelin R (2010). *MEG: An Introduction to Methods*.

[30] Gross J, Baillet S, Barnes GR (2013). Good practice for conducting and reporting MEG research. *Neuroimage*.

[31] Misulis KE, Fakhoury T (2001). *Spehlmann's Evoked Potential Primer*.

[32] Basar E (2012). A review of alpha activity in integrative brain function: fundamental physiology, sensory coding, cognition and pathology. *International Journal of Psychophysiology*.

[33] Tallon-Baudry C, Bertrand O (1999). Oscillatory gamma activity in humans and its role in object representation. *Trends in Cognitive Sciences*.

[34] Bertrand O, Tallon-Baudry C (2000). Oscillatory gamma activity in humans: a possible role for object representation. *International Journal of Psychophysiology*.

[35] Uhlhaas PJ, Singer W (2006). Neural synchrony in brain disorders: relevance for cognitive dysfunctions and pathophysiology. *Neuron*.

[36] Uhlhaas PJ, Singer W (2011). The development of neural synchrony and large-scale cortical networks during adolescence: relevance for the pathophysiology of schizophrenia and neurodevelopmental hypothesis. *Schizophrenia Bulletin*.

[37] Engel AK, Fries P, Singer W (2001). Dynamic predictions: oscillations and synchrony in top-down processing. *Nature Reviews Neuroscience*.

[38] Bartos M, Vida I, Jonas P (2007). Synaptic mechanisms of synchronized gamma oscillations in inhibitory interneuron networks. *Nature Reviews Neuroscience*.

[39] Pfurtscheller G (2001). Functional brain imaging based on ERD/ERS. *Vision Research*.

[40] Jackson JD (1962). *Classical Electrodynamics*.

[41] Sarvas J (1987). Basic mathematical and electromagnetic concepts of the biomagnetic inverse problem. *Physics in Medicine and Biology*.

[42] Tian TS (2010). Functional data analysis in brain imaging studies. *Frontiers in Psychology*.

[43] Katila TE (1983). Round table. On the current multipole presentation of the primary current distributions—Rome, September 15, 1982. *Il Nuovo Cimento D*.

[44] Baillet S, Mosher JC, Leahy RM (2001). Electromagnetic brain mapping. *IEEE Signal Processing Magazine*.

[45] Pascual-Marqui RD, Michel CM, Lehmann D (1994). Low resolution electromagnetic tomography: a new method for localizing electrical activity in the brain. *International Journal of Psychophysiology*.

[46] Uutela K, Hämäläinen M, Somersalo E (1999). Visualization of magnetoencephalographic data using minimum current estimates. *NeuroImage*.

[47] Stenbacka L, Vanni S, Uutela K, Hari R (2002). Comparison of minimum current estimate and dipole modeling in the analysis of simulated activity in the human visual cortices. *NeuroImage*.

[48] Baillet S, Mosher JC, Leahy RM Electromagnetic brain imaging using brainstorm.

[49] Lin F, Witzel T, Ahlfors SP, Stufflebeam SM, Belliveau JW, Hämäläinen MS (2006). Assessing and improving the spatial accuracy in MEG source localization by depth-weighted minimum-norm estimates. *NeuroImage*.

[50] Gramfort A, Kowalski M, Hämäläinen M (2012). Mixed-norm estimates for the M/EEG inverse problem using accelerated gradient methods. *Physics in Medicine and Biology*.

[51] Van Veen BD, Van Drongelen W, Yuchtman M, Suzuki A (1997). Localization of brain electrical activity via linearly constrained minimum variance spatial filtering. *IEEE Transactions on Biomedical Engineering*.

[52] Gross J, Kujala J, Hämäläinen M, Timmermann L, Schnitzler A, Salmelin R (2001). Dynamic imaging of coherent sources: studying neural interactions in the human brain. *Proceedings of the National Academy of Sciences of the United States of America*.

[53] Hashizume A, Iida K, Shirozu H (2007). Gradient magnetic-field topography for dynamic changes of epileptic discharges. *Brain Research*.

[54] Nenonen J Magnetic Source Imaging.

[55] Enatsu R, Mikuni N, Usui K (2008). Usefulness of MEG magnetometer for spike detection in patients with mesial temporal epileptic focus. *NeuroImage*.

[56] Mohseni HR, Smith PP, Parsons CE (2012). MEG can map short and long-term changes in brain activity following deep brain stimulation for chronic pain. *Plos ONE*.

[57] Hari R, Salmelin R, Tissari SO, Kajola M, Virsu V (1994). Visual stability during eyeblinks. *Nature*.

[58] Bardouille T, Picton TW, Ross B (2006). Correlates of eye blinking as determined by synthetic aperture magnetometry. *Clinical Neurophysiology*.

[59] Despopoulos A, Silbernagel S (1991). *Color Atlas of Physiology*.

[60] Jousmäki V, Hari R (1996). Cardiac artifacts in magnetoencephalogram. *Journal of Clinical Neurophysiology*.

[61] Berg P, Scherg BM (1994). A multiple source approach to the correction of eye artifacts. *Electroencephalography and Clinical Neurophysiology*.

[62] Meyer CD (2001). *Matrix Analysis and Applied Linear Algebra Book and Solutions Manual*.

[63] Cardoso J (1998). Blind signal separation: statistical principles. *Proceedings of the IEEE*.

[64] James CJ, Hesse CW (2005). Independent component analysis for biomedical signals. *Physiological Measurement*.

[65] Rong F, Contreras-Vidal JL (2006). Magnetoencephalographic artifact identification and automatic removal based on independent component analysis and categorization approaches. *Journal of Neuroscience Methods*.

[66] Okada Y, Jung J, Kobayashi T (2007). An automatic identification and removal method for eye-blink artifacts in event-related magnetoencephalographic measurements. *Physiological Measurement*.

[67] Mantini D, Franciotti R, Romani GL, Pizzella V (2008). Improving MEG source localizations: an automated method for complete artifact removal based on independent component analysis. *NeuroImage*.

[68] Dammers J, Schiek M, Boers F (2008). Integration of amplitude and phase statistics for complete artifact removal in independent components of neuromagnetic recordings. *IEEE Transactions on Biomedical Engineering*.

[69] Klados MA, Papadelis C, Braun C, Bamidis PD (2011). REG-ICA: a hybrid methodology combining Blind Source Separation and regression techniques for the rejection of ocular artifacts. *Biomedical Signal Processing and Control*.

[70] Escudero J, Hornero R, Abásolo D, Fernández A (2011). Quantitative evaluation of artifact removal in real magnetoencephalogram signals with blind source separation. *Annals of Biomedical Engineering*.

[71] Taulu S, Kajola M (2005). Presentation of electromagnetic multichannel data: the signal space separation method. *Journal of Applied Physics*.

[72] Taulu S, Simola J, Kajola M (2005). Applications of the signal space separation method. *IEEE Transactions on Signal Processing*.

[73] Taulu S, Simola J (2006). Spatiotemporal signal space separation method for rejecting nearby interference in MEG measurements. *Physics in Medicine and Biology*.

[74] Taulu S, Kajola M, Simola J (2004). Suppression of interference and artifacts by the signal space separation method. *Brain Topography*.

[75] Taulu S, Simola J, Kajola M (2004). Clinical applications of the signal space separation method. *Frontiers in Human Brain Topography*.

[76] Medvedovsky M, Taulu S, Bikmullina R, Ahonen A, Paetau R (2009). Fine tuning the correlation limit of spatio-temporal signal space separation for magnetoencephalography. *Journal of Neuroscience Methods*.

[77] Medvedovsky M, Taulu S, Bikmullina R, Paetau R (2007). Artifact and head movement compensation in MEG. *Neurology, Neurophysiology, and Neuroscience*.

[78] Kakisaka Y, Wang ZI, Mosher JC (2012). Clinical evidence for the utility of movement compensation algorithm in magnetoencephalography: successful localization during focal seizure. *Epilepsy Research*.

[79] Nenonen J, Nurminen J, Kicic D (2012). Validation of head movement correction and spatiotemporal signal space separation in magnetoencephalography. *Clinical Neurophysiology*.

[80] Carrette E, De Tiège X, De Beeck MO (2011). Magnetoencephalography in epilepsy patients carrying a vagus nerve stimulator. *Epilepsy Research*.

[81] Litvak V, Eusebio A, Jha A (2012). Movement-related changes in local and long-range synchronization in Parkinson's disease revealed by simultaneous magnetoencephalography and intracranial recordings. *Journal of Neuroscience*.

[82] (2000). *The Merck Manual of Medical Information*.

[83] Karceski S (2008). How to identify the signs and symptoms of partial seizures. *Practical Neurology*.

[84] Knowlton RC, Shih J (2004). Magnetoencephalography in epilepsy. *Epilepsia*.

[85] Stefan H, Rampp S, Knowlton RC (2011). Magnetoencephalography adds to the surgical evaluation process. *Epilepsy and Behavior*.

[86] Burch J, Marson A, Beyer F (2012). Dilemmas in the interpretation of diagnostic accuracy studies on presurgical workup for epilepsy surgery. *Epilepsia*.

[87] Rose DF, Smith PD, Sato S (1987). Magnetoencephalography and epilepsy research. *Science*.

[88] Rose DF, Sato S, Ducla-Soares E, Kufta CV (1991). Magnetoencephalographic localization of subdural dipoles in a patient with temporal lobe epilepsy. *Epilepsia*.

[89] Baumgartner C, Pataraia E, Lindinger G, Deecke L (2000). Neuromagnetic recordings in temporal lobe epilepsy. *Journal of Clinical Neurophysiology*.

[90] Bouet R, Jung J, Delpuech C (2012). Towards source volume estimation of interictal spikes in focal epilepsy using magnetoencephalography. *NeuroImage*.

[91] Madhavan D, Heinrichs-Graham E, Wilson TW (2013). Whole-brain functional connectivity increases with extended duration of focal epileptiform activity. *Neuroscience Letters*.

[92] Stefan H, Schneider S, Feistel H (1992). Ictal and interictal activity in partial epilepsy recorded with multichannel magnetoelectroencephalography: correlation of electroencephalography/electrocorticography, magnetic resonance imaging, single photon emission computed tomography, and positron emission tomography findings. *Epilepsia*.

[93] Ray A, Bowyer SM (2010). Clinical applications of magnetoencephalography in epilepsy. *Annals of Indian Academy of Neurology*.

[94] Eliashiv DS, Elsas SM, Squires K, Fried I, Engel J (2002). Ictal magnetic source imaging as a localizing tool in partial epilepsy. *Neurology*.

[95] Sutherling WW, Crandall PH, Engel J (1987). The magnetic field of complex partial seizures agrees with intracranial localizations. *Annals of Neurology*.

[96] Fujiwara H, Greiner HM, Hemasilpin N (2012). Ictal MEG onset source localization compared to intracranial EEG and outcome: improved epilepsy presurgical evaluation in pediatrics. *Epilepsy Research*.

[97] Medvedovsky M, Taulu S, Gaily E (2012). Sensitivity and specificity of seizure-onset zone estimation by ictal magnetoencephalography. *Epilepsia*.

[98] Paulini A, Fischer M, Rampp S (2007). Lobar localization information in epilepsy patients: MEG—a useful tool in routine presurgical diagnosis. *Epilepsy Research*.

[99] Heers M, Rampp S, Stefan H (2012). MEG-based identification of the epileptogenic zone in occult peri-insular epilepsy. *Seizure*.

[100] Iwasaki M, Pestana E, Burgess RC, Lüders HO, Shamoto H, Nakasato N (2005). Detection of epileptiform activity by human interpreters: blinded comparison between electroencephalography and magnetoencephalography. *Epilepsia*.

[101] Knake S, Halgren E, Shiraishi H (2006). The value of multichannel MEG and EEG in the presurgical evaluation of 70 epilepsy patients. *Epilepsy Research*.

[102] Paulini A (2006). Lobar localisation information: comparison of EEG and MEG. *Epilepsia*.

[103] Abou-Khalil B (2007). An update on determination of language dominance in screening for epilepsy surgery: the Wada test and newer noninvasive alternatives. *Epilepsia*.

[104] Baxendale S (2009). The Wada test. *Current Opinion in Neurology*.

[105] Carlson C (2010). Wada you do for language: fMRI and language lateralization?. *Epilepsy Currents*.

[106] Papanicolaou AC, Simos PG, Castillo EM (2004). Magnetocephalography: a noninvasive alternative to the Wada procedure. *Journal of Neurosurgery*.

[107] Frye RE, Rezaie R, Papanicolaou AC (2009). Functional neuroimaging of language using magnetoencephalography. *Physics of Life Reviews*.

[108] Doss RC, Zhang W, Risse GL, Dickens DL (2009). Lateralizing language with magnetic source imaging: validation based on the Wada test. *Epilepsia*.

[109] Hirata M, Goto T, Barnes G (2010). Language dominance and mapping based on neuromagnetic oscillatory changes: comparison with invasive procedures: clinical article. *Journal of Neurosurgery*.

[110] Billingsley-Marshall RL, Clear T, Mencl WE (2007). A comparison of functional MRI and magnetoencephalography for receptive language mapping. *Journal of Neuroscience Methods*.

[111] Marinkovic K, Dhond RP, Dale AM, Glessner M, Carr V, Halgren E (2003). Spatiotemporal dynamics of modality-specific and supramodal word processing. *Neuron*.

[112] McDonald CR, Thesen T, Hagler DJ (2009). Distributed source modeling of language with magnetoencephalography: application to patients with intractable epilepsy. *Epilepsia*.

[113] Pirmoradi M, Béland R, Nguyen DK, Bacon BA, Lassonde M (2010). Language tasks used for the presurgical assessment of epileptic patients with MEG. *Epileptic Disorders*.

[114] Wellmer J, Weber B, Urbach H, Reul J, Fernandez G, Elger CE (2009). Cerebral lesions can impair fMRI-based language lateralization. *Epilepsia*.

[115] Frith U (2003). *Autism: Explaining the Enigma*.

[116] Volkmar FR, Lord C, Bailey A, Schultz RT, Klin A (2004). Autism and pervasive developmental disorders. *Journal of Child Psychology and Psychiatry and Allied Disciplines*.

[117] Baron-Cohen S, Belmonte MK (2005). Autism: a window onto the development of the social and the analytic brain. *Annual Review of Neuroscience*.

[118] Caronna EB, Milunsky JM, Tager-Flusberg H (2008). Autism spectrum disorders: clinical and research frontiers. *Archives of Disease in Childhood*.

[119] Shen Y, Dies KA, Holm IA (2010). Clinical genetic testing for patients with autism spectrum disorders. *Pediatrics*.

[120] Newschaffer CJ, Croen LA, Daniels J (2007). The epidemiology of autism spectrum disorders. *Annual Review of Public Health*.

[121] Neville BGR (1999). Magnetoencephalographic patterns of epileptiform activity in children with regressive autism spectrum disorders. *Pediatrics*.

[122] Kanner AM (2000). Commentary: the treatment of seizure disorders and EEG abnormalities in children with autistic spectrum disorders: are we getting ahead of ourselves?. *Journal of Autism and Developmental Disorders*.

[123] Rapin I (2006). Language heterogeneity and regression in the autism spectrum disorders—overlaps with other childhood language regression syndromes. *Clinical Neuroscience Research*.

[124] Neville BGR (1999). Magnetoencephalographic patterns of epileptiform activity in children with regressive autism spectrum disorders. *Pediatrics*.

[125] Kallen RJ, Lewine JD, Davis JT (2001). A long letter and an even longer reply about autism magnetoencephalography and electroencephalography. *Pediatrics*.

[126] Muñoz-Yunta JA, Ortiz T, Palau-Baduell M (2008). Magnetoencephalographic pattern of epileptiform activity in children with early-onset autism spectrum disorders. *Clinical Neurophysiology*.

[127] Sasaki M, Nakagawa E, Sugai K (2010). Brain perfusion SPECT and EEG findings in children with autism spectrum disorders and medically intractable epilepsy. *Brain and Development*.

[128] Dawson G, Webb SJ, McPartland J (2005). Understanding the nature of face processing impairment in autism: insights from behavioral and electrophysiological studies. *Developmental Neuropsychology*.

[129] Happé F, Frith U (2006). The weak coherence account: detail-focused cognitive style in autism spectrum disorders. *Journal of Autism and Developmental Disorders*.

[130] Elgar K, Campbell R, Skuse D (2002). Are you looking at me? Accuracy in processing line-of-sight in Turner syndrome. *Proceedings of the Royal Society B*.

[131] Hadjikhani N, Joseph RH, Snyder J, Tager-Flusberg H (2007). Abnormal activation of the social brain during face perception in autism. *Human Brain Mapping*.

[132] Jemel B, Mottron L, Dawson M (2006). Impaired face processing in autism: fact or artifact?. *Journal of Autism and Developmental Disorders*.

[133] Sun L, Grützner C, Bolte S (2012). Impaired gamma-band activity during perceptual organization in adults with autism spectrum disorders: evidence for dysfunctional network activity in frontal-posterior cortices. *Journal of Neuroscience*.

[134] Mooney CM, Ferguson GA (1951). A new closure test. *Canadian Journal of Psychology*.

[135] Roberts TPL, Schmidt GL, Egeth M (2008). Electrophysiological signatures: magnetoencephalographic studies of the neural correlates of language impairment in autism spectrum disorders. *International Journal of Psychophysiology*.

[136] Braeutigam S, Swithenby SJ, Bailey AJ (2008). Contextual integration the unusual way: a magnetoencephalographic study of responses to semantic violation in individuals with autism spectrum disorders. *European Journal of Neuroscience*.

[137] Nishitani N, Avikainen S, Hari R (2004). Abnormal imitation-related cortical activation sequences in Asperger's syndrome. *Annals of Neurology*.

[138] Happé F (1999). Autism: cognitive deficit or cognitive style?. *Trends in Cognitive Sciences*.

